# Topical formulations containing *Copaifera duckei* Dwyer oleoresin improve cutaneous wound healing 

**Published:** 2021

**Authors:** Fernanda Gosuen Gonçalves Dias, Lucas de Freitas Pereira, Ricardo Andrade Furtado, Geórgia Modé Magalhães, Marina Pacheco Miguel, Luis Gustavo Gosuen Gonçalves Dias, Adriana Torrecilhas Jorge, Cristiane dos Santos Honsho, Sérgio Ricardo Ambrósio, Jairo Kenupp Bastos, Micaela Silva Carrijo, Denise Crispim Tavares

**Affiliations:** 1 *Department of Veterinary Medicine, Universidade de Franca, Franca (SP), Brazil*; 2 *Department of Veterinary Medicine, Instituto Federal do Sul de Minas Gerais, Muzambinho (MG), Brazil*; 3 *Department of Veterinary Medicine, Universidade Federal de Goiás, Goiânia (GO), Brazil *; 4 *Department of Veterinary Medicine, Universidade Estadual Paulista, Jaboticabal (SP), Brazil *; 5 *Department of Sciences, Universidade de Franca, Franca (SP), Brazil*; 6 *Department of Pharmacy, Universidade de São Paulo, Ribeirão Preto (SP), Brazil*

**Keywords:** Cutaneous wound healing, Copaifera duckei Dwyer oleoresin, Toxicity

## Abstract

**Objective:**

Evaluation of the healing and toxicological effects of *Copaifera duckei* Dwyer oleoresin (CDO).

**Materials and Methods:**

Rodents with skin lesions were divided into nine groups, including daily treatments with 1, 3 and 10% CDO, collagenase, antibiotic ointment and control groups, for 14 days.

**Results:**

Treatment with 10% CDO reduced skin edema and hyperplasia, demonstrating anti-inflammatory effect of the oil. Reduction in the wound area was observed, indicating the healing effect of CDO. Histopathological analysis showed increases in angiogenesis and re-epithelialization in animals treated with the highest concentration. On the other hand, no alterations in ulcerations, inflammatory infiltrate, hemorrhage, congestion, degeneration, percentage of collagen fibers, number of cells stained with anti-macrophage migration inhibitory factor, or density of area stained with anti-collagen I and III were found. Toxicogenetic analysis revealed no differences in micronucleus frequencies or in the ratio of polychromatic erythrocytes to total erythrocytes between treated and negative control, demonstrating the absence of genotoxicity and cytotoxicity, respectively. There was no difference in levels of liver enzymes among groups, indicating the absence of hepatotoxicity.

**Conclusion:**

Formulations of CDO exerted beneficial effects on the stages of cutaneous wound healing and are promising options for the treatment of wounds.

## Introduction

Due to its extension and anatomical characteristics, the skin is subject to different harmful stimuli, including mechanical, thermal (heat, chemical or electricity), chemical or biological ones. In this sense, skin wounds from burns have a high rate of occurrence and, according to Haagsma et al. (2016) ▶, 67 million people worldwide were affected in 2015, resulting in 176,000 deaths. These researchers also reported that the high incidence of this type of skin lesion in developing countries, can be attributed to the lack of protection for those affected.

Skin lesions can directly affect natural physiology and partially or totally, destroy the skin and its attachments. The severity of the injury can vary with intensity and time of exposure to the injury. Depending on the depth and location of the damage, adjacent tissues are affected, compromising cutaneous homeostasis and putting the patient's life at risk. In response to tissue discontinuity, the body triggers alternative metabolic pathways in an attempt to re-establish lost functions (Martin et al., 2013 ▶).

Similarly, the process of cutaneous healing comprises a complex cascade and commercially available conservative treatment options for superficial skin wounds are designed to promote rapid coaptation of wound edges, prevent secondary infections, reduce the deleterious effects of microorganisms, minimize local sensitivity, prevent wound progression and complications, and reestablish tissue function and esthetics. However, investigation of new medications in dermatology, especially those containing natural products, is justifiable (Sun et al., 2011 ▶).

The use of medicinal plants in the process of skin repair and healing has increased considerably, especially those with antibacterial, healing, and analgesic activities and those that can induce acceleration in the formation of epithelialization and granulation tissue (Riella et al., 2012 ▶). Therefores, several natural products have already been topically tested for this purpose and have shown varying satisfactory results, such as *Camellia sinensis*, propolis (Vieira et al., 2008), *Aloe vera* (Ramos and Pimentel, 2011 ▶), *Chenopodium ambrosioides,* *Lippia gracilis* (Sérvio et al., 2011 ▶),* Brassica oleracea* (Rebolla et al., 2013 ▶), *Ruta graveolens *(Pistore et al., 2014 ▶), *Triticum sp.* (Tillmann et al., 2014 ▶) and *Annona muricata* (Moghadamtousi et al., 2015 ▶).

Within this context, *Copaifera duckei *Dwyer (family Leguminosae and subfamily Caesalpinoideae) is an option because of the anti-inflammatory, analgesic and antibacterial effects of its oleoresin (Santos et al., 2013 ▶). Considering the medicinal properties of copaiba oil, the large number of cutaneous wounds and lack of literature data on the use of this oleoresin in dermatology, this study evaluated the effect of topical formulations containing *C. duckei* Dwyer oleoresin (CDO) on the healing of experimentally induced superficial cutaneous wounds through clinical and histopathological examination, as well as genotoxic, cytotoxic and hepatotoxic parameters. 

## Materials and Methods

***Copaifera duckei*** **Dwyer oleoresin**

Collected in Belém, PA, Brazil (01º06.933’ S latitude and 48º19.781’ W longitude), the plant material exsiccates were submitted to botanical identification (NID: 96/2012) in the Embrapa Herbarium in Belém (PA). The yield of the volatile fraction of the oleoresin, which is mainly composed of sesquiterpenes, was 27% and non-volatile fraction, which is mainly composed of diterpenes, was 73%. Diterpenes ent-polyaltic acid, ent-dihydroagathic acid, ent-agathic acid, ent-kaurenoic acid, and ent-agathic acid-15-methyl ester were identified by ultraviolet and mass spectrometry-high performance liquid chromatography (Xevo^®^ TQ-S Waters Corporation, Milford, MA, USA) and sesquiterpenes α-bergamotene, β-caryophyllene, β-elemene, α-guaiene, α-humulene, and β-selinene by mass spectrometry-gas chromatography (Shimadzu do Brasil^®^ - QP 2010, São Paulo, SP, Brazil).

**Preparation of the topical formulations**

Lanette N (polyoxyethylene alcohol plus sodium alkyl sulfate; Basf S.A.^®^, São Paulo, SP, Brazil), supplemented with hard paraffin (Solven Solventes e Químicos Ltda., Hortolândia, SP, Brazil), Nipagim (methylparaben; Sulfal Química Ltda., Belo Horizonte, MG, Brazil), Nipazol (propylparaben; Codossal Química Ltda., Recife, PE, Brazil), propylene glycol (All Chemistry, São Paulo, SP, Brazil) and deionized water, was used as excipient to form the anionic base cream. Using the same excipient, the collagen cream (from *Clostridium histolyticum*; Sigma-Aldrich, St. Louis, MO, USA) was prepared as the reference drug. The CDO was added to the anionic base cream at final concentrations of 1, 3 and 10%.

**Superficial wound induction**

Male Wistar rats (*Rattus norvegicus*) were supplied by the Animal House of the School of Pharmaceutical Sciences of Ribeirão Preto, University of São Paulo (Ribeirão Preto, SP, Brazil). The animals were kept in controlled conditions of temperature (22±2°C) and humidity (50±10%) under a 12 hr light-dark cycle, with standard rat chow and water available *ad libitum*. The study was approved by the Animal Care and Use Committee of the University of Franca (process n^o^. 059/15).

The animals were anesthetized by intraperitoneal administration of ketamine hydrochloride (50 mg/kg; Agener União Saúde Animal, Embu-Guaçu, SP, Brazil) and xylazine hydrochloride (5 mg/kg; Konig, Mairinque, SP, Brazil) (Flecknell et al., 2007 ▶). A circular skin fragment (20 mm in diameter and 0.2 mm in depth) was excised using a biopsy punch (Carson and Hladik, 2009 ▶). 

**Experimental design**

The rats were divided into nine groups of six animals each: control with cutaneous injury (CI); cutaneous injury treated with the anionic base cream (IAC); cutaneous injury treated with the topical formulations containing 1, 3 and 10% *C. duckei* Dwyer oleoresin (CDO 1%, CDO 3% and CDO 10%, respectively); cutaneous injury treated with collagenase (ICOL), and cutaneous injury treated with antibiotic ointment (IAO, Nebacetin^®^: 5 mg neomycin sulfate and 250 IU zinc bacitracin; Takeda Farmacêutica Brasil, São Paulo, SP, Brazil). The treatments were done blindly. Negative (no treatment-NC) and methyl methanesulfonate (MMS, Sigma-Aldrich, St. Louis, MO, USA; 40 mg/kg body weight [b.w.], a single intraperitoneal dose) control groups were included for the toxicogenetic assays (OECD 474, 2016).

Wounds were cleaned by sodium chloride solution (Brasmédica S.A. Indústria Farmacêutica Ltda., São Paulo, SP, Brazil). The groups with injury (except for CI) were treated with 300 g of the topical formulations for 14 days. The layer of the formulation was covered with a sterile gauze (Cremer SA, São Paulo, SP, Brasil) and the dressing was attached to a self-adhesive porous bandage (Cobam 3M^TM^, Saint Paul, Minnesota, EUA). Tramadol hydrochloride (5 mg/kg, subcutaneous, every 12 hr up to postoperative day 7 - Cristália Produtos Químicos e Farmacêuticos Ltda, São Paulo - SP, Brazil) was applied as analgesic (Flecknell et al., 2007 ▶).

At the end of the experiment, the animals were euthanized by sodium pentobarbital (120 mg/kg b.w., intraperitoneal; Cristália Produtos Químicos e Farmacêuticos Ltda., São Paulo, SP, Brazil) (Flecknell et al., 2007 ▶). Blood and bone marrow samples were collected immediately for biochemical and toxicogenetic analyses, respectively.

**Clinical examination**

**Immediate**

The animals were examined daily to identify behavioral alterations, dermatitis adjacent to the primary injury resulting from the topical products, and other manifestations that could reflect instantaneous discomfort to the applications.

**During topical treatment**

The observers were blind to the treatment condition. Skin edema and hyperplasia was evaluated by scores 4, 8 and 14 days (T4, T8 and T14) after wound induction using the following scoring: 0 (absent), 1 (mild), 2 (moderate) or 3 (intense).

For analysis of the progression of healing compared to time 0 (T0), i.e., immediately after wound induction, and consequently the percent reduction in wound size after treatment, the injuries were photographed by a digital camera at a distance of 10 cm and analyzed using the ImageJ^® ^software (ImageJ/Fiji^®^ 1.50b - (http://rsbweb.nih.gov/ij/) for automatic calculation of the wound area in mm^2^.

**Histopathological analysis**

The skin, including any portion that was still injured and an intact margin of 5 mm up to the musculature, was removed. Histological slides were prepared and stained with hematoxylin-eosin (HE), Gomori trichrome and picrosirius (all from Sigma-Aldrich, St. Louis, MO, USA). 

The HE-stained slides were evaluated under bright-field microscope (40x) for semi-quantitative analysis of angiogenesis, ulceration, re-epithelization, inflammatory infiltration, hemorrhage and congestion and scored as 0 (absent), 1 (mild), 2 (moderate) or 3 (intense). Collagen fiber degeneration was analyzed by staining with Gomori trichrome under bright-field microscope using the same criteria as for the HE-stained slides. Picrosirius staining evaluated the structural distribution and birefringence pattern of collagen fibers and their percentage was quantified by the ImageJ^®^ program.

Three samples of each fragment were mounted on slides with a positive load for immunohistochemical detection of macrophage migration inhibitory factor (MIF) (1 μl: 400 μl, Abcam ab7207 - Spring Bioscience Reveal Biotin-Free System - Roche^®^, Basileia, Suíça), collagen type I (1 μl: 800 μl, Abcam ab90395 - Spring Bioscience Reveal Biotin-Free System - Roche^®^, Basileia, Suíça), and collagen type III (1 μl: 200 μl, Abcam ab7778 - Spring Bioscience Reveal Biotin-Free System - Roche^®^, Basileia, Suíça). For analysis of anti-MIF staining, positive cells were counted in 15 photomicrographed fields of the wound (area of 18.73 mm^2^ each) at 40x magnification. Cells exhibiting a brown staining and an identifiable nucleus were counted using the ImageJ^®^ program and the mean number of counted cells in the 15 fields, was calculated. For evaluation of anti-collagen I and III staining, the density of the stained area was determined in five photomicrographed fields of the wound (area of 323.15 mm^2 ^each) at 100x magnification. The area of brown staining was determined using the ImageJ^® ^program. The mean percentage of this stained area in the five fields and the total area in mm^2^ was calculated.

**Toxicogenetic assessment**

The genotoxic and cytotoxic potential was evaluated in bone marrow (OECD, 474). For genotoxicity, 5,000 polychromatic erythrocytes (PCEs) were analyzed per animal to obtain the frequency of micronucleated polychromatic erythrocytes (PCEMNs). For evaluation of cytotoxicity, 500 erythrocytes/animal were analyzed and the ratio of PCEs/total erythrocytes was determined.

**Biochemical tests**

Liver enzymes [aspartate (AST) and alanine (ALT) aminotransferases] were measured in an automatic biochemical analyzer (Mindray BS-200, Shenzhen, China) using enzymatic colorimetric kits (Dialab, Belo Horizonte, MG, Brazil). 

**Statistical analysis**

The results were analyzed by simple analysis of variance (ANOVA) for randomized experiments, calculating F statistics and the respective p-value. In case of p≤0.05, treatment means were compared by the Tukey test and the least significant difference was calculated for α=0.05 (GraphPad Prism^®^). 

## Results

**Clinical**

**Immediate**

None of the animals exhibited behavioral alterations or clinical signs of immediate discomfort to the treatments.

**During topical treatments**

**Edema**

**Figure 1:**
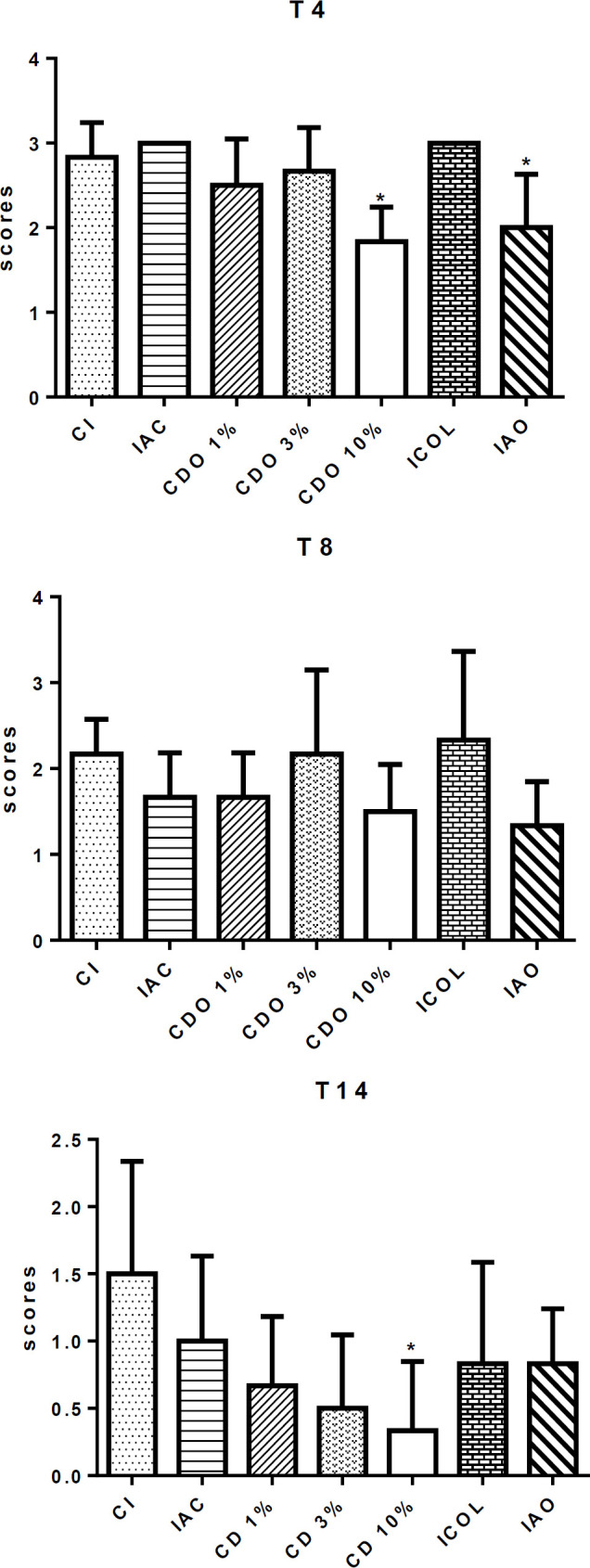
Scores of edema in cutaneous wounds induced on the back of rats treated with formulations of *Copaifera duckei* oleoresin and their respective controls

On T4 and T14, edema was significantly lower in CDO 10% compared to the CI (p<0.05), demonstrating possible anti-inflammatory effects of CDO at the highest concentration. CDO 1% and CDO 3% did not significantly reduce edema. Similar results were observed in IAC, ICOL and IAO, except for IAO on T4 (Figure 1).

**Skin hyperplasia**

**Figure 2:**
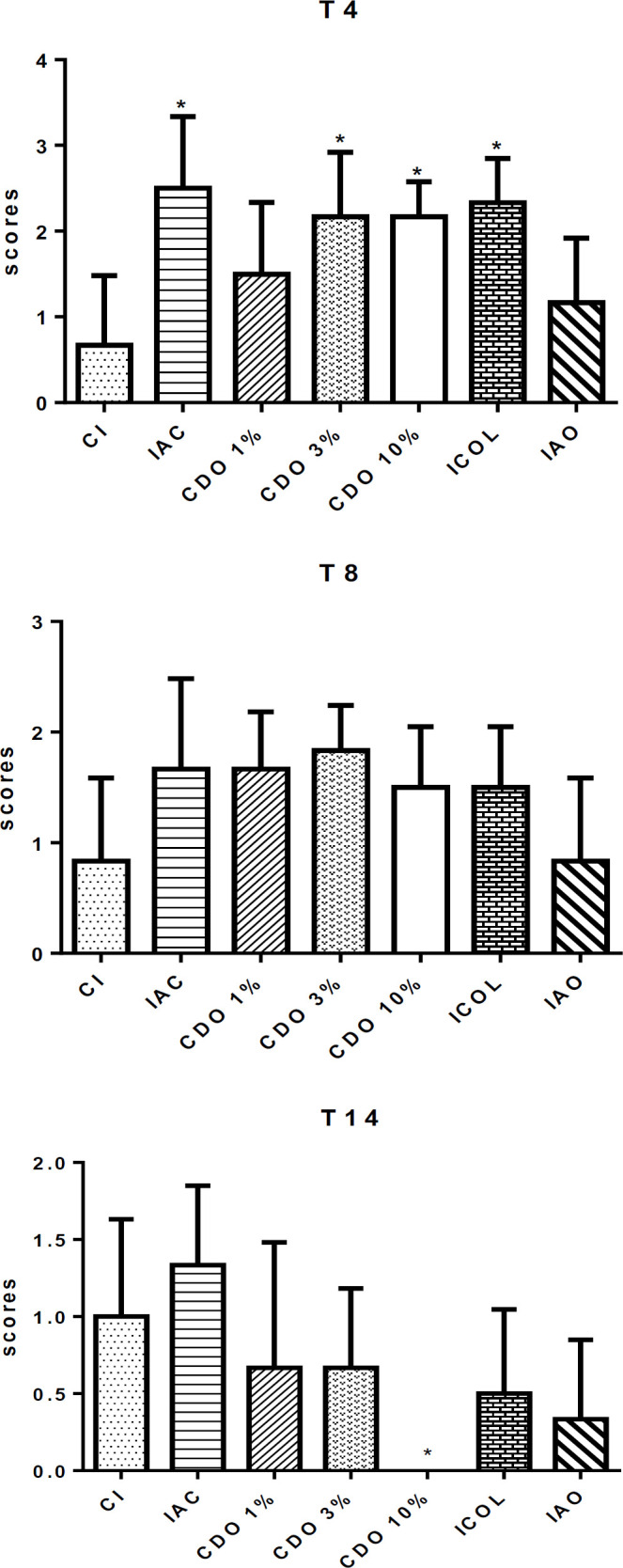
Scores of skin hyperplasia in cutaneous wounds induced on the back of rats treated with formulations of *Copaifera duckei* oleoresin and their respective controls

On T4, only CDO 1% did not result in a significant difference when compared to CI. On T8, no difference was observed among groups, indicating that the different concentrations of CDO did not negatively affect the early stages of wound healing, which require the occurrence of an inflammatory process. The skin hyperplasia was significantly reduced on T14 of the CDO 10% when compared to the other treatments (p<0.05), including the groups treated with the conventional drugs (Figure 2). 

**Wound area**

On T4, the CDO 10% had significantly smaller areas than those of the CI (p<0.05), while there was no difference between the groups treated with the commercial drugs and CI. On T8, a significant reduction was observed in the CDO 10% group when compared to T0 and to the CI (p<0.05). A similar result was found for ICOL. 

T4, T8 and T14: treated for 4, 8 and 14 days, respectively; CI: untreated group; IAC: group treated with anionic base cream; CDO 1%, CDO 3% and CDO 10%: groups treated with formulations containing 1%, 3% and 10% of *C. duckei *oleoresin, respectively; ICOL: group treated with collagenase ointment; IAO: group treated with Nebacetin^®^.

Values are mean±standard deviation.

^*^Significantly different from CI at the respective time of treatment (p<0.05).

T4, T8 and T14: treated for 4, 8 and 14 days, respectively; CI: untreated group; IAC: group treated with anionic base cream; CDO 1%, CDO 3% and CDO 10%: groups treated with formulations containing 1%, 3% and 10% of *C. duckei *oleoresin, respectively; ICOL: group treated with collagenase ointment; IAO: group treated with Nebacetin^®^. Values are mean±standard deviation.

^*^Significantly different from CI at the respective time of treatment (p<0.05).

**Figure 3:**
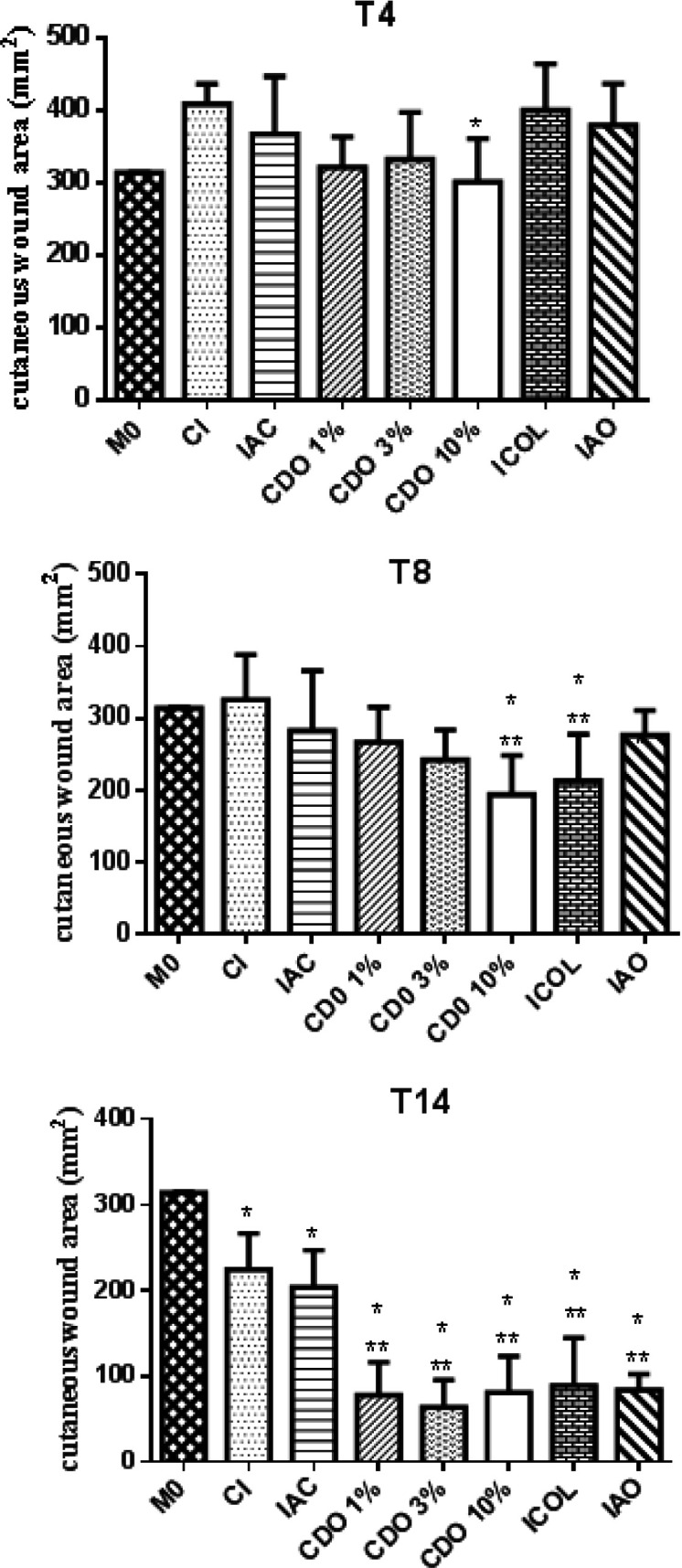
Area (in mm^2^) of wounds induced on the back of rats treated with formulations of *Copaifera duckei* oleoresin and their respective controls

There was a significant decrease in the areas on T14 in all groups compared to T0 (p<0.05). In addition, the groups treated with CDO and the commercial drugs exhibited a significant decrease in the wounds on T14 when compared to CI (p<0.05), suggesting a healing effect for CDO, especially at the highest concentration (Figure 3).

T4, T8 and T14: treated for 4, 8 and 14 days, respectively; CI: untreated group; IAC: group treated with anionic base cream; CDO 1%, CDO 3% and CDO 10%: groups treated with formulations containing 1%, 3% and 10% of *C. duckei *oleoresin, respectively; ICOL: group treated with collagenase ointment; IAO: group treated with Nebacetin^®^. Values are mean±standard deviation.

^*^Significantly different from CI at the respective time of treatment (p<0.05).

^**^Significantly different from T0 (P<0.05).

**Histopathological**

**Hemorrhage, congestion and inflammatory infiltrate**

**Figure 4:**
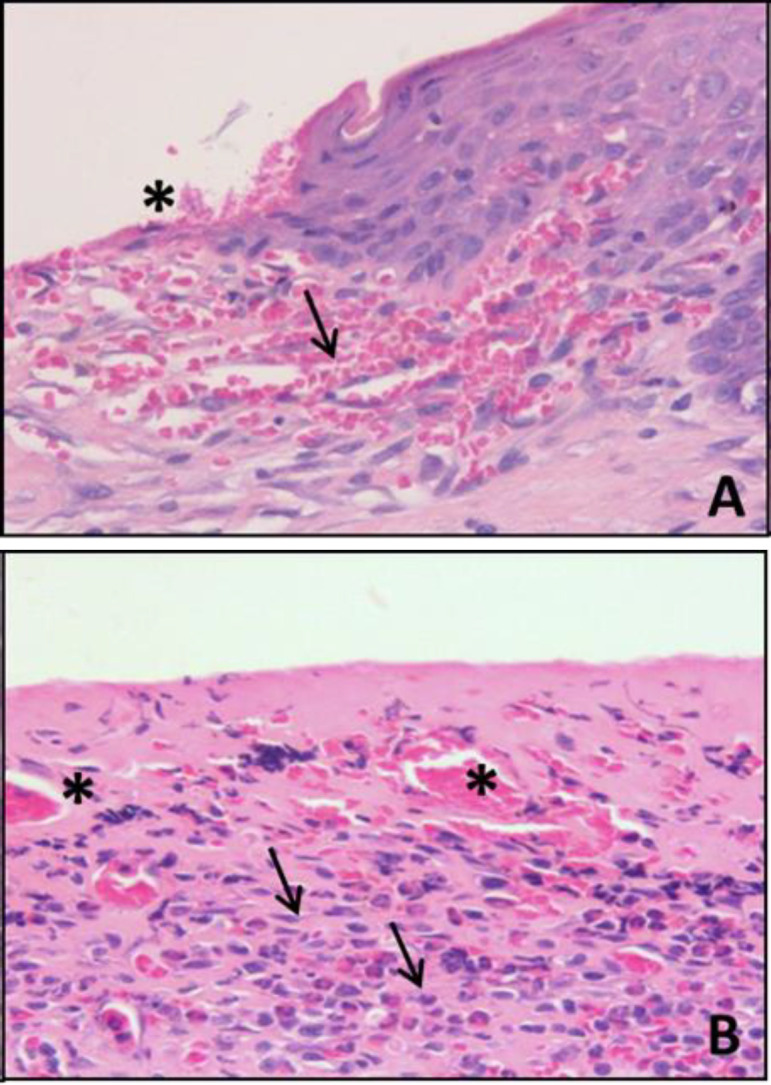
Digital photomicrographs of the skin of rats (HE, 40x) showing an area of moderate ulceration (*) and mild hemorrhage (arrow) in group CDO 3% (A), and moderate congestion (*) and an intense number of inflammatory cells (arrows) in an ulcerated area of the group CDO 1% (B)

**Figure 5:**
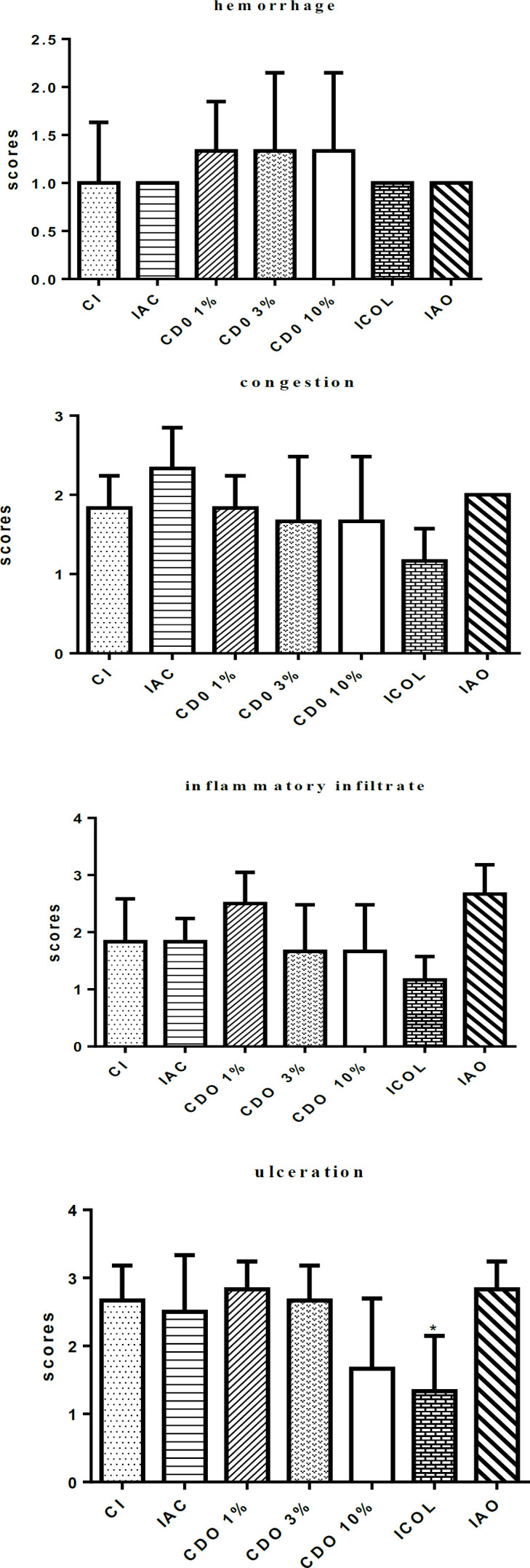
Scores of hemorrhage, congestion, inflammatory infiltrate and ulceration in wounds induced on the back of rats treated for 14 days with formulations of *Copaifera duckei* oleoresin and their respective controls. CI: untreated group; IAC: group treated with anionic base cream; CDO 1%, CDO 3% and CDO 10%: groups treated with formulations containing 1%, 3% and 10% of *C. duckei *oleoresin, respectively; ICOL: group treated with collagenase ointment; IAO: group treated with Nebacetin^®^. Values are mean±standard deviation

^*^Significantly different from CI (p<0.05).

Comparison with the CI showed no significant differences in the parameters studied for any of the groups, including IAO, nor between treatment groups (Figures 4 and 5). 

**Ulceration**

There was no significant reduction in CDO 1%, 3% and 10% when compared to CI. The reductions were significant only for ICOL (p<0.05) (Figure 5).

**Collagen degeneration**

**Figure 6:**
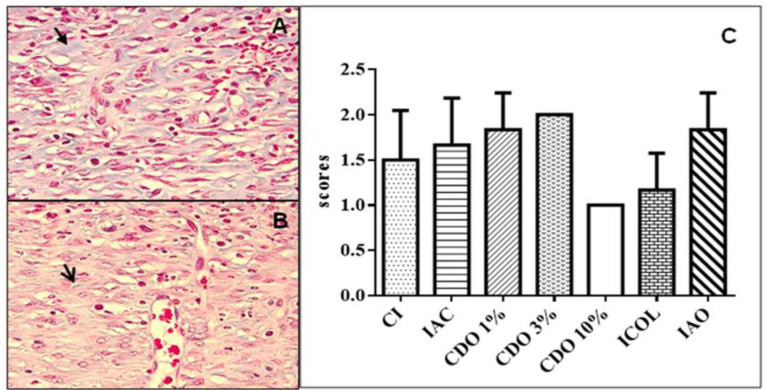
A. Digital photomicrograph of the skin of a rat with poor collagen degeneration. Note the maintenance of blue staining (arrow) in an animal of the CDO 10% group. B. moderate collagen degeneration. Little blue staining (arrow) in the CDO 3% group (Gomori trichrome, 40x). C. Scores of collagen degeneration intensity

CI: untreated group; IAC: group treated with anionic base cream; CDO 1%, CDO 3% and CDO 10%: groups treated with formulations containing 1%, 3% and 10% of *C. duckei *oleoresin, respectively; ICOL: group treated with collagenase ointment; IAO: group treated with Nebacetin^®^.

No significant difference was observed for any of the groups when compared to CI, even ICOL and IAO (Figure 6).

**Angiogenesis**

**Figure 7:**
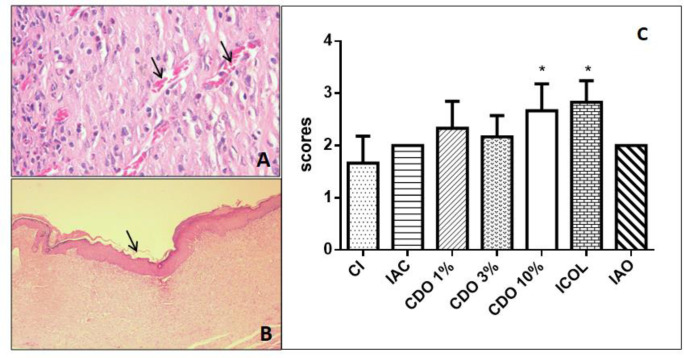
A. Digital photomicrograph of the skin of a rat treated with CDO 1% showing an area of intense angiogenesis (arrows). B. Rat treated with CDO 10% showing moderate re-epithelization in the wound area (arrow) (HE, 40x). C. Scores of angiogenesis intensity in wounds of rats treated for 14 days with formulations of *Copaifera duckei* oleoresin and their respective controls

CI: untreated group; IAC: group treated with anionic base cream; CDO 1%, CDO 3% and CDO 10%: groups treated with formulations containing 1%, 3% and 10% of *C. duckei *oleoresin, respectively; ICOL: group treated with collagenase ointment; IAO: group treated with Nebacetin^®^.

The CDO 10% exhibited a significant increase in new vessels compared to CI (p<0.05), which probably contributed to the acceleration of healing. Similar results were observed for ICOL, without a significant difference compared to CDO 10% (Figure 7).

**Macrophage migration inhibitory factor**

Statistical comparison showed no significant difference in the number of anti-MIF-positive cells between the CDO 1%, 3%, and 10% and CI. There were also no significant differences for the other treatment groups compared to CI.

Values are mean±standard deviation.

Values are mean±standard deviation.^*^Significantly different from CI (p<0.05).

**Percentage of collagen**

The formulations containing CDO did not differ significantly from CI in terms of their collagen-stimulating properties. Similar results were observed for ICOL and IAO. 

Regarding collagen type I, there were no significant differences between the CDO 1%, 3%, and 10% and CI. Similar results were found for ICOL and IAO. No significant differences between the treatment groups and CI were observed for collagen type III. 

**Re-epithelization**

**Figure 8:**
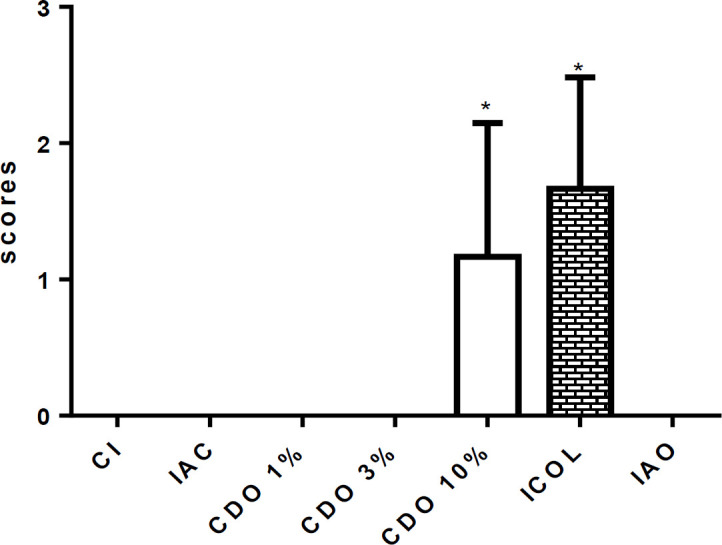
Scores of re-epithelization intensity in wounds induced on the back of rats treated for 14 days with formulations of *Copaifera duckei* oleoresin and their respective controls

CI: untreated group; IAC: group treated with anionic base cream; CDO 1%, CDO 3% and CDO 10%: groups treated with formulations containing 1%, 3% and 10% of *C. duckei *oleoresin, respectively; ICOL: group treated with collagenase ointment; IAO: group treated with Nebacetin^®^. Values are mean±standard deviation.

^*^Significantly different from CI (p<0.05).

The re-epithelization was significantly increased in the CDO 10% compared to the CI (p<0.05), that probably favored and accelerated healing of animals treated with the highest concentration tested. Similar results were observed for the ICOL, without a significant difference compared to CDO 10%. The other treated groups did not show the same results, including IAO (Figure 8).

**Toxicogenetic assessment**

No significant difference in the frequency of PCEMNs or in the PCE/total erythrocyte ratio was observed between the treatment groups and the NC (no treatment), demonstrating the absence of genotoxicity and cytotoxicity, respectively.

**Biochemical analysis**

There was no significant difference in ALT or AST levels for any of the groups compared to the NC, indicating the absence of hepatotoxicity. 

## Discussion

The pharmaceutical form of CDO was based on texture (lower viscosity and easier dispersion), removal of cell debris during dressing changes and in the favoring of the moisture in the wound bed (Bathia et al., 2014 ▶). Lanette N acts as a colloidal disperser, conferring emollience and softness to the skin. Nipagim and Nipazol protect the formulations against bacteria, fungi and yeast and propylene glycol functions as a humectant. Thus, after manipulation, the formulations did not exhibit physical alterations like the formation of crystals or lumps, color changes or contamination. Studies that incorporated CDO into topical formulations did not specify the excipients used (Lima et al., 2011 ▶; Carvalho et al., 2015 ▶), a fact that limits comparisons of the results.

In the present study, edema reduction at the wound edges was observed in CDO 10%, 4 and 14 days after surgical induction, suggesting an anti-inflammatory property for the oleoresin. This effect might be attributed to the sesquiterpenes, particularly β-caryophyllene (Veiga Junior et al., 2007 ▶; Leandro et al., 2012 ▶). On the other hand, Brito et al. (1999) ▶ found greater inflammation in cutaneous wounds treated with the oil of *C. reticulata* compared to those treated with saline, probably for the use in natura of the oleoresin.

Skin hyperplasia observed in all groups up to T8 is due to the constitutive production of proinflammatory cytokines that are necessary for the early stages of tissue repair (Santiago et al., 2015 ▶). The results showed that the oleoresin did not negatively interfere with the proinflammatory process at any of the concentrations tested. At the end of the experimental period, a significant reduction was observed in the group CDO 10% compared to the other, including those treated with the conventional drugs. This reduction can be explained, at least in part, by the anti-inflammatory property of the oleoresin, attributed to constituents sesquiterpenes and diterpenes (Macri et al., 2014 ▶). 

The group CDO 10% showed significantly reduced wound areas at the three time points studied, demonstrating a healing effect for the oleoresin at this concentration. On the other hand, Vieira et al. (2008) who tested the oleoresin from *C. langsdorffii* in surgical wounds of rats reported divergent results, probably because they used a coverslip in the wound. The foreign body in injured tissues causes macrophages to stop expressing their beneficial functions during repair and to exert phagocytic activity, interrupting the sequence of tissue regeneration events.

Histopathologic analysis of re-epithelization also indicated the healing effect of the CDO 10%, corroborating with the clinical examination of the wound areas. The formulations did not interfere with fibrin formation or with the activation of pro-aggregatory factors and platelets. These elements favor the production of growth factors that induce keratinocytes proliferation and migration to the wound edges, reorganizing adhesion molecules and stimulating granulation tissue (Reinke and Sorg, 2012 ▶). Furthermore, the oleoresin may have contributed to the activation of genes in viable cells adjacent to the site of injury, which are essential for tissue re-epithelization (Reinke and Sorg, 2012 ▶). In addition to this property of the copaiba oil used in this study, there are the advantages of the preconized dressing, especially debridement and daily cleaning. 

Histologically, the CDO 10% exhibited a significant increase in the number of new blood vessels, which contributed to wound re-epithelization. According to Gibot et al. (2020) ▶, angiogenesis favors the oxygenation of injured tissues and triggers the development of granulation tissue by contributing to the chemotaxis of inflammatory mediators. At this concentration, the oleoresin may have stimulated substances that exert a signaling function in basement membrane cells of the newly formed capillaries (Ahmed et al., 2015 ▶). The functions of inflammatory cells include control of microorganisms and removal of cell debris from the wounds. The transport of these cells to the site of injury depends on neovascularization (Rebolla et al., 2013 ▶). Taken together, the present results suggest that, by inducing angiogenesis, CDO favored the migration of mono- and polymorphonuclear cells. 

According to Martin et al. (2013) ▶, the persistence of inflammatory cells in the wound for long periods, may compromise re-epithelization because of the release of proteolytic enzymes that damage the still healthy adjacent tissue. However, although no significant reduction in these cells occurred on T14 in groups CDO, no interference with re-epithelization was observed. Furthermore, inflammatory cells, especially neutrophils, release growth factors (trephones) that stimulate the production of fibroblasts and consequently collagen (Carvalho et al., 2005 ▶). In this respect, collagen was detected in the groups evaluated but there was no significant difference among treatments. In agreement with these results, Carvalho et al. (2005) ▶, who evaluated the effect of the *C. duckei* Dwyer oleoresin on dermatitis and limb edema in rats demonstrated anti-inflammatory activity on both types of lesion. Studying human peripheral blood, Santiago et al. (2015) ▶ described an inhibitory effect for the oleoresin from *C. duckei* and *C. reticulata* on the excessive production of proinflammatory interleukins, which can compromise healing. In that study, the authors emphasized the balancing effect of the two copaiba species on the production of pro- and anti-inflammatory cytokines. 

Despite the advantages of the commercial ointment, the animals of the IAO group exhibited results similar to those treated with the CDO formulations, in terms of wound hemorrhage and congestion, as well as clinical (skin hyperplasia) and histopathological parameters (angiogenesis, re-epithelization, inflammatory infiltrate, and collagen deposition). Thus, the CDO formulations are a promising alternative for the conservative treatment of superficial cutaneous wounds.

In addition to fibroblasts, extracellular matrix components and collagen fibers, growth factors are fundamental to skin reconstitution. Collagen is a protein synthesized by fibroblasts and, to a lesser extent, by endothelial cells (Reinke and Sorg, 2013 ▶). Collagen was evaluated in the present study because it is one of the main structural components of a scar and the extracellular matrix that confers tensile strength and elasticity to the skin. Immunohistochemistry revealed no effect for the CDO formulations on the presence of different types of collagen, suggesting that their healing effect is related to inflammation and angiogenesis. In this regard, the extract of *Annona muricata* was also shown to accelerate healing due to its anti-inflammatory activity (Moghadamtousi et al., 2015 ▶). However, positive effects resulting from a reduction in collagen deposition during skin regeneration, were demonstrated in a study evaluating the oleoresin from *C. reticulata*, suggesting that this plant subproduct can exert collagenolytic activity (Brito et al., 1999 ▶).

Collagenase is the main proteolytic enzyme capable of digesting wound collagen filaments but does not affect the protein of intact tissue (Reinke and Sorg, 2012 ▶). Thus, inclusion of the ICOL group permitted to compare the possible enzymatic effects of the treatments proposed. The results showed no difference in quantitative or qualitative collagen analysis between animals treated with the CDO formulations in comparison with the CI group, as observed in the treatment with the reference drug (collagenase). 

MIF is a multifunctional protein widely expressed in skin. The involvement of MIF in skin repair is related to functions such as a proinflammatory in the immune response (Emmerson et al., 2009 ▶), proliferation, fibroplasia, scar contracture, and angiogenesis. Within this context, MIF mediates crucial events such as growth, migration, suppression of apoptosis, maintenance and inactivation of cell cycle inhibitors (Gilliver et al., 2011 ▶). Zhao et al. (2005) reported that MIF accelerates the healing of cutaneous wounds induced in the dorsal skin of rodents. In the present study, MIF staining was observed in all groups; however, there was no significant difference among them, including animals treated with the conventional drugs, demonstrating that the topical CDO formulations did not influence the expression of this protein.

The CDO formulations showed no hepatoxicity, in agreement with Hajiaghaalipour et al. (32) who reported that topical medications generally have low toxicity. Lima et al. (2011) ▶ evaluated the effects of *C. duckei* Dwyer oleoresin applied as an ingredient of a vaginal cream or as a natural product and observed no elevation of ALT or AST levels for the two pharmaceutical forms. On the other hand, Noguchi et al. (2002) ▶ found reduction in these enzymes when they tested the oleoresin from *C. reticulata* administered by gavage to rodents for 5 days. These findings corroborate the study of Castro-e-Silva Júnior et al. (2004) ▶ who identified an antiproliferative activity against hepatocytes during liver regeneration in rats after the administration of *C. duckei* Dwyer. According to the authors, this oleoresin may alter the mitochondrial function of hepatocytes and promote changes in liver function. To evaluate the adverse effects of the topical CDO formulations on non-target organs of the study, we analyzed their toxicogenetic potential by the micronucleus test. The results revealed the absence of genotoxic or cytotoxic effects, corroborating the findings of Furtado et al. (2018) ▶. 

The results suggest that the CDO formulations, especially the highest concentration, favor some stages of superficial wound healing similar to conventional drugs and are promising options in these conditions. In addition, the formulations did not cause genotoxicity or hepatotoxicity. Further studies should be conducted to better understand the molecular signaling pathways involved in the CDO wound healing action, such as the investigation of the involvement of epidermal growth factors, as well as enzymatic antioxidants in skin repair.
